# Contrast-Associated Acute Kidney Injury After Thrombectomy for Ischemic Stroke: Prognostic Impact and CAN-REST Predictive Score

**DOI:** 10.1212/WNL.0000000000214655

**Published:** 2026-02-19

**Authors:** Ghil Schwarz, Angelo Cascio Rizzo, Gareth Ambler, Pawel Wrona, Agnieszka Slowik, Szymonn Kotas, Mohamed F. Doheim, Alhamza R. Al-Bayati, Raul G. Nogueira, Ana Paiva Nunes, Patricia Ferreira, Matteo Paolucci, Andrea Zini, Luigi Simonetti, Norbert Leško, Jakub Fedorko, Zuzana Gdovinova, Luca Scarcia, Erwah Kalsoum, Firas Farhat, Morin Beyeler, Adnan Mujanovic, Marcel Arnold, Torcato Meira, Marta Morais, Leonor Francisco, Marcin Wiacek, Paulina Pudło, Halina Bartosik-Psujek, Patricia Calleja, Fernando Ostos, David Seoane, Anca Negrila, Razvan Alexandru Radu, Cristina Tiu, Sami Al Kasab, Ahmad Abu Qdais, Imad Samman Tahhan, Oscar Ayo-Martin, Maria Paya, Juan David Molina-Nuevo, Beata Labuz-Roszak, Danilo Toni, Karolina Moszko, Mateusz Łukasz Roszak, Manuela De Michele, Elena Barrile, Prasanna Eswaradass, Margaret Houghton, Tiffany Barkley, Amir Ali, Jelle Demeestere, Wayne Martin Bauknight, Sunil Sheth, Louise Maes, Anke Wouters, João Pedro Marto, Josè Blazer Costa, Nicola D. Loizzo, Andrea Romi, Carmelo Tiberio Currò, Piotr Luchowski, Maksymilian Seweryn, Konrad Rejdak, Piers Klein, Mohamad Abdalkader, Valentina Saia, Antioco Sanna, Tiziana Tassinari, Maurizio Acampa, Francesca Rosini, Rossana Tassi, Marta Bilik, Anna Maria Bandzarewicz-Samcik, Jiangyong Min, Naser HajAissa, Marilena Mangiardi, Sabrina Anticoli, Enrico Pampana, Carlos Hervás-Testal, Ricardo Rigual, Blanca Fuentes, Davide Strambo, Carl Manuata Tetaria, Guillaume Saliou, Andrea Salvatore Caramma, Davide Maimone, Pier Andrea Rizzo, Marco Moci, Irene Scala, Antonio Cruz-Culebras, Rocío Vera Lechuga, Sebastián García-Madrona, Giovanni Merlino, Mariarosaria Valente, Arianna Cella, Manuel Bolognese, Lehel-Barna Lakatos, Grzegorz M. Karwacki, Paolo Candelaresi, Vincenzo Andreone, Carlo Maurea, Roberto Tarletti, Angelica Mele, Antonio Ciacciarelli, Michele Alessiani, Gabriella Monteforte, David Pakizer, Martin Roubec, David Školoudík, Fenne Vandervorst, Sylvie De Raedt, Martijn Verdam, Josef Bartoš, Martin Sramek, Valentina Mazzoleni, Luca Quilici, Dario Alimonti, Jessica Moller, Ilaria Maestrini, Marina Diomedi, Giordano Lacidogna, Osama O. Zaidat, Eugene Lin, Mohammad Almajali, Claudio Baracchini, Matteo Zaccagnino, Federica Viaro, Alessandro Pezzini, Giulia Avola, Chiara Ferraro, Milena Świtońska, Paulina Sobieszak-Skura, Grzegorz Meder, Lukas Mayer-Suess, Michael Knoflach, Elke R. Gizewski, Matthew R. Common, Patrick Nicholson, Sarah Power, Marta Nowakowska-Kotas, Michal Puła, Maciej Guzinski, Soledad Pérez-Sánchez, Joan Montaner, Alexandra Sevilla Bravo, Simona Marcheselli, Francesca Vodret, Costanza Maria Rapillo, Liliana Pereira, Miguel Rodrigues, Adam Jaros, Martin Kovar, Jan Vojik, Gloria Valcamonica, Serena Gallo Cassarino, Alessandra Cardillo, Marco Petruzzellis, Silvia Grimaldi, Nicola Marrone, Ameer E. Hassan, Samantha Miller, Mohammad W. Khasawneh, Valentina Poretto, Simone Tonello, Saverio Tollot, Aleksander DęBiec, Jacek Staszewski, Adam Stepien, Francisco Bernardo, Jorge Ferrao, Marialuisa Zedde, Rosario Pascarella, Alessandra Sanna, Rossella Meloni, Sennur Delibas, Simona Sacco, Maria Grazia Vittorini, Raffaele Ornello, Marina Mannino, Valeria Terruso, Marco Filizzolo, Emily Hall, Christine Roffe, Malgorzata Dorobek, Justyna Zielińska-Turek, Giuseppe Scopelliti, Leonardo Pantoni, Guglielmo C. Pero, Antonio Macera, Amedeo Cervo, Simone Bellavia, Mine Sezgin, Yavuz Dilmen, Esme Ekizoglu, Georgios Tsivgoulis, Aikaterini Theodorou, Vlad Tiu, Cristina Aura Panea, Simona Petrescu, Mariangela Piano, Thanh N. Nguyen, Maria Sessa, as the CAN-REST Study Group

**Affiliations:** 1 Stroke Center, Service of Neurology, Department of Clinical Neurosciences, University Hospital of Lausanne and University of Lausanne, Switzerland;; 2 Department of Neurology and Stroke Unit - ASST Grande Ospedale Metropolitano Niguarda, Milan, Italy;; 3 Department of Statistical Science, University College London, United Kingdom;; 4 Department of Neurology, Jagiellonian University Medical College, Krakow, Poland;; 5 Student Scientific Group in Cerebrovascular Diseases, Faculty of Medicine, Jagiellonian University Medical College, Krakow, Poland;; 6 Stroke Institute, Department of Neurology, University of Pittsburgh Medical Center, PA;; 7 Stroke Unit, Hospital de São José, Unidade Local de Saúde de São José, Lisboa, Portugal;; 8 Centro Clínico Académico de Lisboa, Portugal;; 9 IRCCS Istituto delle Scienze Neurologiche di Bologna, UOC Neurologia e Rete Stroke Metropolitana, Ospedale Maggiore;; 10 IRCCS Istituto Delle Scienze Neurologiche di Bologna, Emilia-Romagna, Italy;; 11 Department of Neurology, Faculty of Medicine, P.J. Safarik University and L. Pasteur University Hospital, Košice, Slovakia;; 12 Department of Radiology, Faculty of Medicine, P.J. Safarik University and L. Pasteur University Hospital, Košice, Slovakia;; 13 Department of Neuroradiology, AP-HP, Henri Mondor University Hospital, DMU FIxIT, Créteil, France;; 14 Department of Neurology, Inselspital, Bern University Hospital, and University of Bern, Switzerland;; 15 Institute for Diagnostic and Interventional Neuroradiology, Inselspital, Bern University Hospital, and University of Bern, Switzerland;; 16 Department of Neuroradiology, Hospital de Braga, Portugal;; 17 School of Medicine, University of Minho, Portugal;; 18 Department of Neurology, Unidade Local de Saúde do Alto Minho, Viana do Castelo, Portugal;; 19 Department of Neurology, Institute of Medical Sciences, Medical College of Rzeszow University;; 20 University of Rzeszow, Poland;; 21 Department of Neurology and Stroke Centre, Hospital Universitario 12 de Octubre, Instituto de Investigación Hospital 12 de Octubre (i+12), Madrid, Spain;; 22 Department of Neurology and Stroke Centre, Hospital Universitario 12 de Octubre, Madrid, Spain;; 23 Stroke Unit, Department of Neurology, University Emergency Hospital Bucharest, Romania;; 24 Neurology Department, Carol Davila University of Medicine and Pharmacy, Bucharest, Romania;; 25 Department of Neurology and Neurosurgery - Medical University of South Carolina;; 26 Department of Neurology, Medical University of South Carolina, Charleston;; 27 Department of Neurosurgery, Medical University of South Carolina, Charleston;; 28 Department of Neurology, Complejo Hospitalario Universitario de Albacete;; 29 Department of Radiology at Complejo Hospitalario Universitario de Albacete;; 30 Department of Neurology, St. Jadwiga Provincional Specialist Hospital, Institute of Medicine, University of Opole, Poland;; 31 Department of Human Neurosciences, Sapienza University of Rome, Italy;; 32 Student Scientific Society at the Department of Neurology, Institute of Medicine, University of Opole, Poland;; 33 Stroke Unit, Emergency Department, Policlinico Umberto I Hospital, Sapienza University of Rome, Italy;; 34 The University of Kansas Medical Center, Kansas City;; 35 The University of Kansas Health System, Kansas City;; 36 McGovern Medical School, UTHealth Houston;; 37 Department of Neurosciences, Experimental Neurology, Leuven Brain Institute, KU Leuven, Belgium;; 38 Department of Neurology, University Hospitals Leuven, Belgium;; 39 Department of Neurology, Hospital de Egas Moniz, Centro Hospitalar Lisboa Ocidental, Portugal;; 40 UO Neurologia d'Urgenza e Stroke Unit, IRCCS Fondazione Mondino, Pavia, Italy;; 41 UOC Neuroradiologia, IRCCS Policlinico San Matteo, Pavia, Italy;; 42 UO Neurologia d'Urgenza e Stroke Unit, IRCCS Fondazione Mondino, Pavia, Italy;; 43 Dipartimento di Scienze del Sistema Nervoso e del Comportamento, Università di Pavia, Italy;; 44 Department of Neurology and Neurological Nursing, Medical University of Lublin, Poland;; 45 Student Scientific Society, Chair and Department of Neurology, Medical University of Lublin, Poland;; 46 Department of Neurology, Medical University of Lublin, Poland;; 47 Neurology, Boston Medical Center, Chobanian and Avedisian School of Medicine, Boston University, MA;; 48 Radiology, Boston Medical Center, Chobanian and Avedisian School of Medicine, Boston University, MA;; 49 Department of Neurology & Stroke Unit, Santa Corona Hospital, Pietra Ligure (SV), Italy;; 50 Neuroradiology Unit, Santa Corona Hospital, Pietra Ligure (SV), Italy;; 51 Stroke Unit, Department of Medical Sciences, Surgery and Neurosciences, University of Siena, Italy;; 52 U.O.C. Stroke Unit, Azienda Ospedaliera Universitaria Senese;; 53 Neurological Ward with Stroke Unit, St John Paul II Western Hospital in Grodzisk Mazowiecki, Faculty of Medicine, Lazarski University, Warszawa, Poland;; 54 Department of Neurosciences, Corewell Health West and Michigan State University, Grand Rapids;; 55 UOSD Stroke Unit, S. Camillo-Forlanini Hospital, Italy;; 56 Department of Neurology and Stroke Centre, La Paz Institute for Health Research - IdiPAZ (La Paz University Hospital - Autonomous University of Madrid), Spain;; 57 Neuroradiology Unit, Service of Diagnostic and Interventional Radiology, Department of Medical Radiology, University Hospital of Lausanne and University of Lausanne, Switzerland;; 58 A.O.E. Cannizzaro di Catania, Italy;; 59 Department of Neuroscience, Catholic University of the Sacred Heart, Rome, Italy;; 60 Hospital Universitario Ramon y Cajal, Madrid, Spain;; 61 Stroke Unit, Udine University Hospital, Italy;; 62 Clinical Neurology, Udine University Hospital, Italy;; 63 DMED, Udine University Hospital, Italy;; 64 Department of Neurology, Cantonal Hospital of Lucerne, Switzerland;; 65 Department of Radiology and Nuclear Medicine, Luzerner Kantonsspital, University Teaching and Research Hospital, University of Lucerne, Switzerland;; 66 AORN Antonio Cardarelli, UOC Neurologia e Stroke Unit;; 67 SCDU Neurologia - Stroke Unit, Azienda Ospedaliero-Universitaria “Maggiore della Carità”, Novara, Italy;; 68 Neurology Division, Santa Maria Goretti Hospital, Latina, Italy;; 69 Centre for Health Research, Department of Clinical Neurosciences, Faculty of Medicine, University of Ostrava, Czech Republic;; 70 Comprehensive Stroke Center, Department of Neurology, University Hospital Ostrava, Czech Republic;; 71 Department of Neurology, Universitair Ziekenhuis Brussel, Belgium;; 72 NEUR Research Group, Center for Neurosciences (C4N), Vrije Universiteit Brussel (VUB), Belgium;; 73 Department of Radiology, Universitair Ziekenhuis Brussel, Belgium;; 74 Military Faculty Hospital, Prague, Czech Republic;; 75 Stroke Unit, Department of Neurology, Prague, Czech Republic;; 76 SC Neurologia - ASST Papa Giovanni XXIII, Bergamo, Italy;; 77 SC Radiologia Diagnostica per Immagini 2 - Neuroradiologia ASST Papa Giovanni XXIII, Bergamo, Italy;; 78 SC Neurologia Stroke Unit, Dipartimento Neurologico e della Riabilitazione, ARNAS G. Brotzu, Cagliari, Italy;; 79 Stroke Center, Department of Systems Medicine, University of Rome “Tor Vergata”, Italy;; 80 Mercy St. Vincent Medical Center, Toledo, OH;; 81 Stroke Unit and Neurosonology Laboratory, Department of Neuroscience, Padua University Hospital, Italy;; 82 Department of Medicine and Surgery, University of Parma, Italy;; 83 Stroke Care Program, Department of Emergency, Parma University Hospital, Italy;; 84 Department of Medicine and Surgery, Neurology Clinic, University of Parma, Italy;; 85 Department of Neurology and Clinical Neurophysiology, Collegium Medicum in Bydgoszcz, Nicolaus Copernicus University in Torun, Poland;; 86 Clinic of Neurology and Clinical Neurophysiology, Jan Biziel University Hospital, Poland;; 87 Department of Interventional Radiology, Jan Biziel University Hospital No. 2, Bydgoszcz, Poland;; 88 Department of Neurology, Medical University of Innsbruck, Austria;; 89 Department of Radiology, Medical University of Innsbruck, Austria;; 90 Department of Neuroradiology, Beaumont Hospital, Dublin, Ireland;; 91 Department of Radiology, Royal College of Surgeons of Ireland, Dublin;; 92 Clinical Department of Neurology, University Centre of Neurology and Neurosurgery, Faculty of Medicine, Wroclaw Medical University, Poland;; 93 Department of General Radiology, Interventional Radiology, and Neuroradiology, Wroclaw Medical University, Poland;; 94 Stroke Unit, Department of Neurology, Hospital Universitario Virgen Macarena, Sevilla, Spain;; 95 IRCCS Humanitas Research Hospital, Rozzano, Italy;; 96 Hospital Garcia de Orta, Unidade Local de Saúde de Almada-Seixal, Portugal;; 97 Department of Neurology, Na Homolce Hospital, Prague, Czech Republic;; 98 Department of Neurology, ASST Ovest Milanese, Legnano, Italy;; 99 Stroke Unit-UOC Neurologia “Puca” - AOU Consorziale Policlinico Bari, Italy;; 100 Department of Neurology, University of Texas Rio Grande Valley - Valley Baptist, Harlingen;; 101 Department of Neurology, Washington University in St. Louis, MO;; 102 Neurology Unit, Department of Neuro-Cardiovascular Medicine, Treviso, Italy;; 103 Neuroradiology, Department of Neuro-Cardiovascular Medicine, Treviso, Italy;; 104 Military Institute of Medicine - National Research Institute, Warsaw, Poland;; 105 Neurology Department and Stroke Unit, Hospital de Faro, ULS Algarve, Farro, Portugal;; 106 Neurology Unit, Stroke Unit, Azienda Unità Sanitaria Locale-IRCCS di Reggio Emilia, Italy;; 107 Neuroradiology Unit, Ospedale Santa Maria della Misericordia, AULSS 5 Polesana, Rovigo, Italy;; 108 SSD Stroke Unit, AOU Sassari, Italy;; 109 Neurology and Interventional Neurology, Antalya Training and Research Hospital, University of Health Sciences, Turkey;; 110 Department of Biotechnological and Applied Clinical Sciences, University of L'Aquila, L'Aquila, Italy;; 111 Department of Neurology and Stroke Unit, AOOR Villa Sofia-V. Cervello, Palermo, Italy;; 112 Department of Radiology, AOOR Villa Sofia-V. Cervello, Palermo, Italy;; 113 Department of Neuroradiology, S. Bortolo Hospital, Vicenza, Italy;; 114 Department of Medicine and Surgery, University Hospitals of North Midlands NHS Trust, Staffordshire, United Kingdom;; 115 Keele University School of Medicine, Staffordshire, United Kingdom;; 116 Department of Neurology, National Institute of Medicine of the Ministry of Interior and Administration, Warsaw, Poland;; 117 Neurology Unit, Luigi Sacco University Hospital, Milan, Italy;; 118 Neuroscience Research Center, Department of Biomedical and Clinical Sciences, University of Milan, Italy;; 119 Department of Neurorehabilitation Sciences, Casa di Cura Igea, Milan, Italy;; 120 Department of Neuroradiology - ASST Grande Ospedale Metropolitano Niguarda, Milan, Italy;; 121 Department of Neurology, Istanbul University Faculty of Medicine, Turkey;; 122 Second Department of Neurology, National & Kapodistrian University of Athens, “Attikon” University Hospital, Greece; and; 123 Neurology Department, Elias University Emergency Hospital, Bucharest, Romania.

## Abstract

**Background and Objectives:**

Contrast-associated acute kidney injury (CA-AKI) is a potentially preventable complication after exposure to iodinated contrast media. In patients undergoing endovascular thrombectomy (EVT) for acute ischemic stroke (AIS), the incidence and clinical impact are poorly characterized, and no validated prediction tool is currently available. The aim of this study was to assess the incidence and prognostic significance of CA-AKI in EVT-treated patients with AIS and to develop and validate a predictive score.

**Methods:**

A retrospective, multicenter cohort study was conducted involving EVT-treated patients across 73 centers in 16 countries (January–December 2023). Inclusion criteria were age ≥18 years, absence of dialysis, availability of preprocedural and 48-hour postprocedural creatinine levels, and available 90-day follow-up (modified Rankin Scale [mRS] score). The primary outcome was CA-AKI, defined by KDIGO (Kidney Disease: Improving Global Outcomes criteria;creatinine increase ≥0.3 mg/dL or ≥1.5 times baseline, within 48 hours). Secondary outcomes were (1) in-hospital mortality, (2) 90-day mRS score, and (3) 90-day severe disability or death (mRS score >3). Logistic models assessing associations with outcomes accounted for within-center clustering by applying robust standard errors. CA-AKI prediction models were developed across imputed data sets using univariable selection (*p* < 0.20), backward elimination (*p* < 0.05), and coefficient-based scoring after categorization of continuous predictors, with internal validation by bootstrap to obtain optimism-adjusted estimates.

**Results:**

Among 6,638 patients (median age 74 years; 48.7% male), CA-AKI occurred in 326 (4.9%) and was independently associated with in-hospital mortality (adjusted odds ratio [aOR] 2.269; 95% CI 1.615–3.190), higher 90-day mRS scores (adjusted common odds ratio 1.584; 95% CI 1.110–2.258), and 90-day severe disability or death (aOR 1.530; 95% CI 1.057–2.216). A preprocedural risk model including 12 routine clinical variables—sex, ethnicity, arterial hypertension, dyslipidemia, chronic kidney disease, antiplatelet therapy, NIH Stroke Scale score at admission, serum glucose, estimated glomerular filtration rate, hemoglobin, mean arterial pressure, and IV thrombolysis—demonstrated acceptable discrimination (area under the receiver operating characteristic curve 0.710 [95% CI 0.682–0.738]; precision-recall area under the curve 0.13 [95% CI 0.10–0.16]), good calibration (slope 0.870 [95% CI 0.759–0.928]), good overall performance (Brier score 0.045 [95% CI 0.042–0.049]). A second model that included EVT-related variables (e.g., contrast volume) showed similar performances.

**Discussion:**

In this large, international cohort, CA-AKI occurred in approximately 1 in 20 EVT-treated patients with AIS and was independently associated with poor outcomes. A simple preprocedural risk score enables early identification of high-risk individuals and may support preventive strategies.

## Introduction

Diagnostic and therapeutic imaging procedures involving intravascular iodinated contrast media are frequently used across medical disciplines.^1^ However, their use has been linked to acute kidney injury (AKI),^2^ a complication first reported in the 1950s.^3^ Since then, the direct causal role of contrast media in AKI has been debated, given the potential influence of confounding factors.^4^ As a result, the term contrast-induced AKI, which implies direct nephrotoxicity,^5^ has been replaced by contrast-associated AKI (CA-AKI)—a broader term encompassing all AKI occurring shortly after contrast exposure, regardless of causality.^6^

In various settings, CA-AKI has been associated with long-term renal dysfunction, need for dialysis, poor outcomes, and increased mortality.^7^ Consequently, risk prediction and prevention have become clinical priorities, particularly in specialties heavily reliant on contrast-enhanced procedures.^8^ Although the overall risk is low in the general population, patient subgroups (e.g., elderly patients or those with multiple comorbidities) may face a significantly elevated risk due to factors such as preexisting renal impairment, emergency procedures, and high contrast load.^9^

Acute ischemic stroke (AIS) is a leading global cause of death and disability.^10^ The advent of endovascular thrombectomy (EVT) has transformed AIS care, dramatically improving outcomes.^11^ However, EVT-treated patients often receive substantial doses of iodinated contrast during both preprocedural imaging and the intervention. Preprocedural contrast is administered intravenously, whereas intraprocedural contrast is delivered intra-arterially in repeated boluses; the latter has been associated with greater nephrotoxic potential than IV administration.^12^ In addition, these patients frequently exhibit comorbidities and hemodynamic instability, rendering them especially vulnerable to AKI. Despite this, CA-AKI remains underexplored in the context of EVT. Existing literature is limited: the studies are mostly based on single-center reports, with variable incidence rates of CA-AKI after EVT, but they associate its occurrence with worse clinical outcomes, including increased disability and mortality.^13^ It is important to note that no validated risk scores are currently available to predict CA-AKI in this population.

Given the limited evidence on this potentially preventable complication, its presumed low incidence but yet potentially high prognostic impact, and the absence of a validated risk score in EVT-treated stroke patients, we conducted a large, multicenter, real-world cohort study aimed at (1) determining the incidence of CA-AKI; (2) evaluating its prognostic implications; and (3) developing and internally validating a clinically applicable risk prediction score, suitable for use both before and shortly after EVT.

## Methods

### Study Design and Population

This work represents the primary analysis of the Contrast-Associated Nephropathy Risk Evaluation in Acute Ischemic Stroke After Endovascular Thrombectomy (CAN-REST) study (NCT06596603), a retrospective, multicenter initiative specifically designed to investigate CA-AKI in patients with AIS treated with EVT. Consecutive patients with AIS undergoing EVT were included from 73 academic and community stroke centers across 16 countries (Europe and United States; in eMethods) between January 1 and December 31, 2023. Inclusion criteria were as follows: age ≥18 years, presentation with AIS eligible for EVT, availability of baseline and post-EVT creatinine values (within 48 hours), absence of end-stage kidney disease requiring dialysis, and availability of 90-day modified Rankin Scale (mRS) outcome. Patients were also included if they underwent diagnostic angiography with intended EVT (i.e., spontaneous or post-IV thrombolysis [IVT] recanalization) and received contrast.

The reporting of this study adheres to the Transparent Reporting of a Multivariable Prediction Model for Individual Prognosis or Diagnosis guidelines.^14^

### Standard Protocol Approvals, Registrations, and Patient Consents

Institutional review board approval was obtained at each site according to local regulations. The study was approved by the Comitato Etico Territoriale Lombardia 3 (approval number 4658_17.04.202_N_bis). The protocol adhered to the Declaration of Helsinki.

### Study Variables and Outcomes

Standardized data were collected on demographics, vascular risk factors, pre-EVT clinical and laboratory values, imaging findings, EVT procedure details, and 90-day clinical outcomes. Variables were defined per consensus standards, detailed in eMethods.

CA-AKI was defined per Kidney Disease: Improving Global Outcomes (KDIGO) criteria as an increase in serum creatinine ≥0.3 mg/dL, or of ≥1.5 times baseline, within 48 hours after contrast administration.^15^

For the association analysis between CA-AKI and clinical outcomes, 3 end points were evaluated: (1) in-hospital mortality, defined as death occurring during the index hospitalization; (2) disability at 90 days, assessed as a shift across the mRS score (range 0 [no disability] to 6 [death]); and (3) severe disability or death at 90 days, defined as mRS score >3 (or an increase of ≥1 point in patients with prestroke mRS score >3).

### Statistical Analysis

Continuous variables were summarized as medians with interquartile ranges (IQRs) and categorical variables as counts and percentages. Missing data were handled using multiple imputation by chained equations (10 data sets).

Univariate associations between CA-AKI and study variables were assessed using logistic regression on complete cases (nonimputed data). Associations between CA-AKI and the outcomes of interest were evaluated using either multivariable binary or ordinal logistic regression, adjusted for covariates showing *p* < 0.20 in the corresponding univariate analyses. This more liberal threshold was adopted to minimize the risk of excluding potential confounders. All multivariable models assessing associations with outcomes accounted for potential nonindependence of observations within centers by applying robust standard errors clustered at the center level.^16^

To assess whether the available sample size was adequate for developing the predictive score, we calculated the required number of patients based on a prespecified calibration slope (0.90), assumed outcome prevalence (5%), number of candidate predictors (28), and expected discrimination (area under the receiver operating characteristic curve [AUC] 0.75).^17^ We then checked this using an “exact” simulation-based method.^18^ These approaches yielded required sample sizes of 5,851 and 6,510 patients, respectively.

The predictive models for CA-AKI were developed based on multiple imputed data sets. The modeling strategy comprised univariable selection, multivariable backward elimination, and categorization, with all steps incorporated within a bootstrap internal validation procedure to avoid overly optimistic estimates of performance. All clinically plausible variables were considered as candidate predictors. Variables associated with CA-AKI at *p* < 0.20 in univariable logistic regression were entered into a multivariable logistic regression model, where backward elimination with a retention threshold of *p* < 0.05 was applied. For clinical applicability, continuous predictors retained after elimination were subsequently categorized independently of the outcome, using thresholds defined a priori either from the observed distribution, to ensure balanced and clinically meaningful strata, or from established and widely used clinical classifications (e.g., KDIGO categories for estimated glomerular filtration rate [eGFR], severity strata for NIH Stroke Scale [NIHSS] score). The final logistic regression model was re-estimated on the multiple imputed data sets. Regression coefficients were then scaled by a factor of 10 and rounded to the nearest integer, generating a simplified point-based score. Each patient's risk score corresponded to the sum of points associated with the predictors present. Discrimination was assessed using both the AUC and the precision-recall area under the curve (PR-AUC), the latter providing a complementary evaluation of model performance in the presence of class imbalance.^19^ Calibration was evaluated by the calibration slope and overall accuracy by the Brier score.^20^ All reported values are optimism-adjusted.

Two related models were developed to reflect different decision points. Model 1 included only variables available before EVT, whereas model 2 incorporated all model 1 predictors + EVT-related procedural variables, which were screened separately from pre-EVT predictors using the same univariable and multivariable backward-elimination criteria. EVT variables retained from this process were then added to the finalized, categorized specification of model 1 without reselecting pre-EVT predictors. The augmented model was re-estimated on the multiple imputed data sets and underwent the same bootstrap internal validation, risk categorization, and point-score derivation, as described above.

To enhance clinical applicability, predicted probabilities were translated into risk categories. Patients were then classified into 3 groups according to their estimated risk of CA-AKI: low risk (<10%), moderate risk (10%–29.9%), and high risk (≥30%). Calibration was visualized with (1) a scatterplot of observed vs predicted risk overall and (2) a calibration plot showing observed and predicted risk within the 3 strata. Finally, an integer-score lookup table was constructed, which, for each score value, reports the mean predicted risk, the number of patients, and the corresponding risk category.

All analyses were performed with Stata version 18.0 (StataCorp., College Station, TX), and statistical significance was defined as *p* < 0.05.

### Data Availability

Anonymized data not included in this article are available on reasonable request to qualified investigators, pending review and approval by the CAN-REST Data Steering Committee. The full Stata analysis code used for model development and validation is publicly available at doi.org/10.5281/zenodo.17602284.

## Results

**Figure 1:**
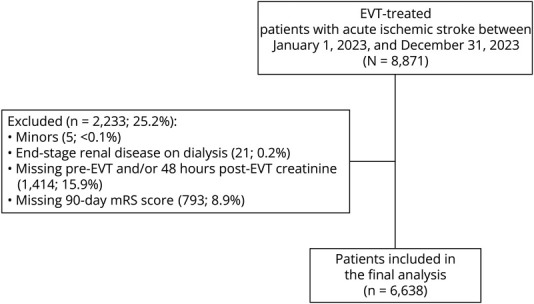
Study Flowchart Overview of patient selection and exclusion criteria leading to the final study cohort. EVT = endovascular thrombectomy.

Between January 1 and December 31, 2023, a total of 8,871 patients underwent EVT across 73 centers. Of these, 2,233 (25.2%) were excluded: 5 were younger than 18 years, 21 had end-stage renal disease requiring dialysis, 1,414 lacked either baseline or within 48-hour post-EVT creatinine measurements, and 793 were lost to 90-day follow-up. The final study population included 6,638 patients, whose baseline and clinical characteristics are summarized in Table 1. The flowchart of patient selection is shown in Figure 1. A comparison between included and excluded patients is provided in eTable 1; imputation details for missing variables are given in eTable 2.

**Table 1:** Baseline Characteristics, Clinical Variables, and Outcomes in the CAN-REST Population, With Univariate Analysis by CA-AKI Status (Nonimputed Data)

	Entire cohort (N = 6,638)	CA-AKI (N = 326 [4.9%])	No CA-AKI (N = 6,312 [95.1%])	*p* Value
Demographics and baseline characteristics				
Age, y, median (IQR)	74 (64–82) [6,638]	75 (65–83) [326]	74 (64–82) [6,312]	0.056
Male sex, n/N (%)	3,231/6,638 (48.7)	170/326 (52.2)	3,061/6,312 (48.5)	0.199
White ethnicity, n/N (%)	5,745/6,192 (92.8)	232/264 (87.9)	5,513/5,928 (93.0)	0.002
BMI	27 (24–30) [4,734]	28 (24–31) [243]	27 (24–30) [4,491]	0.043
Prestroke mRS score, median (IQR)	0 (0–1) [6,556]	0 (0–1) [323]	0 (0–1) [6,233]	<0.001
Medical history and risk factors, n/N (%)				
Known chronic kidney disease	2,604/6,638 (39.2)	198/326 (60.7)	2,406/6,312 (38.1)	<0.001
Arterial hypertension	4,876/6,629 (73.6)	264/325 (81.2)	4,612/6,304 (73.2)	0.001
Diabetes mellitus	1,727/6,631 (26.0)	113/325 (34.8)	1,614/6,306 (25.6)	<0.001
Dyslipidemia	3,318/6,624 (50.1)	151/325 (46.5)	3,167/6,299 (50.3)	0.180
Atrial fibrillation	2,355/6,623 (35.6)	126/324 (38.9)	2,229/6,299 (35.4)	0.199
Coronary artery disease and/or heart failure	1,872/6,620 (28.3)	123/325 (37.9)	1,749/6,295 (27.8)	<0.001
Active cancer	493/6,404 (7.7)	28/314 (8.9)	465/6,090 (7.6)	0.407
Antiplatelets	1,835/6,638 (27.6)	120/326 (36.8)	1,715/6,312 (27.2)	<0.001
Anticoagulants	1,464/6,638 (22.1)	72/326 (22.1)	1,392/6,312 (22.1)	0.989
RAAS inhibitors	2,742/6,432 (42.6)	133/315 (42.2)	2,609/6,117 (42.7)	0.881
Metformin	844/5,827 (14.5)	53/276 (19.2)	791/5,551 (14.3)	0.023
NSAIDs <48 h	331/5,927 (5.6)	22/293 (7.5)	309/5,634 (5.5)	0.143
Acute-phase variables				
Baseline NIHSS score, median (IQR)	15 (10–20) [6,575]	16 (12–20) [325]	15 (10–20) [6,250]	0.002
Mean arterial pressure, median (IQR)	103 (93–115) [6,347]	104 (93–118) [312]	103 (93–115) [6,035]	0.078
Admission blood glucose, mg/dL, median (IQR)	123 (106–151) [6,449]	131 (105–168) [315]	123 (106–150) [6,134]	<0.001
Admission hemoglobin, g/dL, median (IQR)	13.3 (12.0–14.4) [6,580]	13.0 (11.0–14.3)[325]	13.3 (12.0–14.5) [6,255]	<0.001
Baseline eGFR, mL/min, median (IQR)	77 (57–92) [6,638]	65 (41–87) [326]	77 (58–93) [6,312]	<0.001
IV thrombolysis, n/N (%)	2,879/6,637 (43.4)	109/326 (33.4)	2,770/6,311 (43.9)	<0.001
Baseline imaging variables				
Pre-EVT contrast volume, mL, median (IQR)	90 (70–100) [5,029]	80 (60–100) [215]	90 (70–100) [4,814]	0.491
LVO (vs MeVO), n/N (%)	4,856/6,553 (74.1)	254/324 (78.4)	4,602/6,229 (73.9)	0.071
Tandem lesion, n/N (%)	960/6,570 (14.6)	54/324 (16.7)	906/6,246 (14.5)	0.283
Anterior circulation (vs posterior circulation), n/N (%)	5,910/6,552 (90.2)	286/324 (88.3)	5,624/6,228 (90.3)	0.232
ASPECTS/pc-ASPECTS, median (IQR)	9 (8–10) [6,124]	9 (7–10) [301]	9 (8–10) [5,823]	0.065
EVT-related variables				
Onset-to-EVT time, min, median (IQR)	255 (175–410) [6,384]	273 (187–420) [312]	255 (174–410) [6,072]	0.086
EVT procedure duration, min, median (IQR)	40 (23–62) [5,994]	40 (25–66) [296]	39 (23–61) [5,698]	0.098
Number of EVT passes, median (IQR)	1 (1–3) [6,386]	1 (1–3) [317]	1 (1–3) [6,069]	0.930
EVT contrast volume, mL, median (IQR)	80 (50–120) [4,748]	100 (60–140) [205]	80 (50–120) [4,543]	0.035
Procedural hypotension, n/N (%)	613/5,018 (12.2)	39/224 (17.4)	574/4,794 (12.0)	0.015
Successful reperfusion (mTICI ≥ 2b)	5,731/6,411 (89.4)	276/319 (86.5)	5,455/6,092 (89.5)	0.087
Early post-EVT variables, n/N (%)				
Additional contrast exposure (<48 h post-EVT)	700/6,059 (11.6)	35/287 (12.2)	665/5,772 (11.5)	0.727
Early neurologic deterioration	400/6,403 (6.9)	35/309 (11.3)	365/6,094 (6.0)	<0.001
Symptomatic hemorrhagic transformation	351/6,403 (5.5)	42/309 (13.6)	309/6,094 (5.1)	<0.001
Peri-EVT IV hydration				0.091
<500 mL	697/5,562 (12.5)	44/266 (16.5)	653/5,296 (12.3)	
500–1,000 mL	2,305/5,562 (41.4)	99/266 (37.2)	2,206/5,296 (41.7)	
>1,000 mL	2,560/5,562 (46.0)	123/266 (46.2)	2,473/5,296 (46.0)	
Other potential AKI causes	1,108/6,253 (17.7)	106/309 (34.6)	1,002/5,944 (16.9)	<0.001
Outcomes				
Length of hospital stay, d, median (IQR)^a^	8 (4–13) [7,414]	9 (6–15) [227]	8 (5–14) [5,879]	0.038
In-hospital mortality, n/N (%)	902/6,612 (13.6)	113/326 (34.7)	789/6,286 (12.6)	<0.001
90-d mRS score, median (IQR)	3 (1–5) [6,638]	5 (2–6) [326]	3 (1–5) [6,312]	<0.001
Severe disability or death at 90 d, n/N (%)^b^	2,778/6,638 (41.9)	203/326 (62.3)	2,575/6,312 (40.8)	<0.001

Abbreviations: AIS = acute ischemic stroke; ASPECTS = Alberta Stroke Program Early CT Score; BMI = body mass index; CA-AKI = contrast-associated acute kidney injury; eGFR = estimated glomerular filtration rate; EVT = endovascular thrombectomy; IQR = interquartile range; LVO = large vessel occlusion (LVOs included ICA, BA, VA, M1, A1, and P1); MAP = mean arterial pressure; MeVO = medium vessel occlusion; mRS = modified Rankin scale; mTICI = modified Thrombolysis in Cerebral Infarction; NIHSS = NIH Stroke Scale; NSAID = nonsteroidal anti-inflammatory drug; pc-ASPECTS = posterior circulation Acute Stroke Prognosis Early CT Score.

[n] = number of patients with available data.

Early Neurological Deterioration: defined as an increase of ≥4 points in the NIHSS score at 24 hours post-EVT, in the absence of hemorrhagic transformation.

Symptomatic Intracerebral Hemorrhage: defined as any parenchymal hemorrhage associated with a ≥4-point worsening in NIHSS.

Other Potential AKI Causes: included hemodynamic instability, urinary obstruction, nephrotoxic drugs, primary renal disorders, autoimmune conditions, infections, systemic inflammation, and thrombotic or vascular complications. In the absence of standardized diagnostic criteria, these factors were assessed by the treating neurologist at each center and considered present if they occurred between symptom onset and follow-up creatinine measurement.

a Length-of-hospital-stay analysis restricted to patients who survived to discharge.

b Defined as 90-day mRS score >3 (or an increase of ≥1 point in patients with pre-stroke mRS score >3).

### Population Description and Incidence of CA-AKI

The median age of included patients was 74 years (IQR 64–82), and 3,231 (48.7%) were male; 5,745 patients (92.8%) were of White ethnicity; among non-White patients, most were of Black ethnicity (n = 213, 2.8%). Preexisting comorbidities were common: 39.2% had chronic kidney disease, 73.6% had arterial hypertension, 26.0% had diabetes mellitus, and 28.3% had either coronary artery disease or heart failure. The baseline median NIHSS score was 15 (IQR 10–20), and the median eGFR was 77 mL/min/1.73 m^2^. Overall, 74.1% of patients had large vessel occlusion, while the remaining 25.9% had medium vessel occlusion, of which 85.9% were M2 occlusions. Occlusions were predominantly in the anterior circulation (90.2%). The median pre-EVT contrast volume was 90 mL (IQR 70–100 mL). The median EVT contrast volume administered was 80 mL (IQR 50–120).

CA-AKI within 48 hours of contrast administration occurred in 326 patients, yielding a cumulative incidence of 4.9% (95% CI 4.4%–5.5%). Supplementary eTable 3 reports country-specific CA-AKI incidence, contrast volume distributions, and EVT durations. Compared with those without CA-AKI, affected patients were less frequently of White ethnicity (87.9% vs 93.0%, *p* = 0.002) and more likely to have a history of chronic kidney disease (60.7% vs 38.1%, *p* < 0.001), diabetes mellitus (34.8% vs 25.6%, *p* < 0.001), and coronary artery disease or heart failure (37.9% vs 27.8%, *p* < 0.001). They also had higher baseline NIHSS scores (median 16 vs 15, *p* = 0.002), lower admission hemoglobin (13.0 vs 13.3 g/dL, *p* < 0.001), lower eGFR (65 vs 77 mL/min/1.73 m^2^, *p* < 0.001), and higher admission serum glucose (131 vs 123 mg/dL, *p* < 0.001). Patients who developed CA-AKI were also less likely to receive IVT before EVT (33.4% vs 43.9%, *p* < 0.001). Early neurologic deterioration (11.3% vs 6.0%, *p* < 0.001), symptomatic intracerebral hemorrhage (13.6% vs 5.1%, *p* < 0.001), intraprocedural arterial hypotension (17.4% vs 12.0%, *p* = 0.015), and other noncontrast potential AKI triggers (further detailed in eTable 4) (34.6% vs 16.9%, *p* < 0.001) were also more common among patients who developed CA-AKI. The volume of contrast media administered before EVT did not differ significantly between groups (80 mL [IQR 60–100] vs 90 mL [IQR 70–100], *p* = 0.491), whereas patients who developed CA-AKI received a significantly higher contrast volume during EVT (100 mL [IQR 60–140] vs 80 mL [IQR 50–120], *p* = 0.035).

### Association Between CA-AKI and 90-Day Outcomes

**Figure 2:**
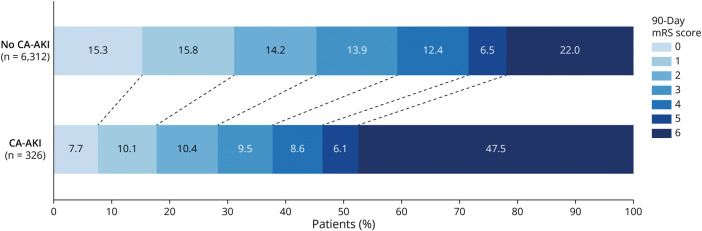
Distribution of 90-Day mRS Scores by CA-AKI Status Grotta bars (stacked proportional bar graphs) showing the distribution of 90-day functional outcomes according to the presence or absence of CA-AKI. CA-AKI = contrast-associated acute kidney injury; mRS = modified Rankin Scale.

In multivariable analyses adjusted for variables with *p* < 0.20 in univariable models (eTables 5–7), CA-AKI remained an independent predictor of worse clinical outcomes (Table 2). Specifically, it was independently associated with in-hospital mortality (adjusted odds ratio [aOR] 2.269; 95% CI 1.615–3.190), higher 90-day mRS score (adjusted common odds ratio 1.584; 95% CI 1.110–2.258), and severe 90-day disability or death (aOR 1.530; 95% CI 1.057–2.216). These findings are illustrated in Figure 2, showing the 90-day mRS score distribution by CA-AKI status.

**Table 2:** Unadjusted and Adjusted Associations Between CA-AKI and Outcomes

Outcomes	Unadjusted OR (95% CI)	*p* Value	Adjusted OR (95% CI)^a^	*p* Value
In-hospital mortality	3.698 (2.428–5.633)	<0.001	2.269 (1.615–3.190)	<0.001
Ordinal shift in 90-d mRS score	2.703 (1.559–4.685)	<0.001	1.584 (1.110–2.258)	<0.001
Severe disability or death at 90 d^b^	2.395 (1.425–4.026)	<0.001	1.530 (1.057–2.216)	0.024

Abbreviations: CA-AKI = contrast-associated acute kidney injury; mRS = modified Rankin Scale; OR = odds ratio.

a Adjusted for variables associated with the outcome at *p* < 0.20 in univariate analysis.

b Defined as 90-day mRS score >3 (or an increase of ≥1 point in patients with prestroke mRS score >3).

### Derivation and Validation of a Predictive Risk Score

The cohort size available for model development was adequate relative to the a priori estimates. Twelve independent predictors of CA-AKI were identified in the preprocedural model (model 1): male sex, non-White ethnicity, arterial hypertension, absence of dyslipidemia, chronic kidney disease, antiplatelet therapy, baseline NIHSS score, baseline serum glucose, estimated GFR, baseline hemoglobin, baseline mean arterial pressure, and direct EVT (i.e., without previous IVT) (Table 3). After converting continuous variables into categorical predictors and assigning weighted integer points, the resulting score-based model demonstrated acceptable discrimination (AUC 0.710 [95% CI 0.682–0.738]; PR-AUC 0.13 [95% CI 0.10–0.16]), strong overall performance (Brier score 0.045 [95% CI 0.0420–0.0486]), and good calibration (slope 0.870 [95% CI 0.759–0.982]). Model 2 included the same preprocedural predictors as model 1, with the addition of EVT contrast agent volume, which was the only procedural variable independently associated with CA-AKI (odds ratio 1.002, 95% CI 1.000–1.004; *p* = 0.031). The score-based model achieved comparable performance to model 1, with an AUC of 0.712 (95% CI 0.684–0.740) and PR-AUC of 0.13 (95% CI 0.10–0.16), a Brier score of 0.045 (95% CI 0.0419–0.0486), and a calibration slope of 0.866 (95% CI 0.756–0.740).

**Table 3:** Predictive Models for CA-AKI: Model 1 (Including Only Pre-EVT Variables) and Model 2 (Including Model 1 + EVT-Related Variables)

	β-coefficient	Integer score	*p* Value	95% CI
Model 1 (pre-EVT): including only pre-EVT variables				
Male sex	0.2617	3	0.033	0.0208 to 0.5025
Non-White ethnicity	0.4216	4	0.031	0.0382 to 0.8049
Arterial hypertension	0.3947	4	0.011	0.0900 to 0.6995
No dyslipidemia	0.3849	4	0.002	0.1458 to 0.6241
Known chronic kidney disease	0.6535	7	0.000	0.4051 to 0.9020
Antiplatelets	0.4025	4	0.001	0.1567 to 0.6482
NIHSS score				
≤10	1 (ref)	0	—	—
>10	0.4061	4	0.005	0.1226 to 0.6896
Glycemia				
<100 mg/dL	0.6059	6	0.001	0.2618 to 0.9499
100–124 mg/dL	1 (ref)	0	—	—
125–149 mg/dL	0.0977	1	0.590	−0.2575 to 0.4529
150–199 mg/dL	0.4093	4	0.022	0.0602 to 0.7583
≥200 mg/dL	0.8896	9	0.000	0.5183 to 1.2608
Baseline estimated GFR				
≥60 mL/min	1 (ref)	0	—	—
30–59 mL/min	0.2788	3	0.060	−0.2974 to 0.5691
15–29 mL/min	1.0130	10	0.000	0.5459 to 1.4801
<15 mL/min	2.0878	21	0.000	1.4065 to 2.7691
Admission hemoglobin				
<10	0.6698	7	0.001	0.2913 to 1.0483
10–11.9	0.2127	2	0.195	−1.1090 to 0.5343
12–13.9	1 (ref)	0	—	—
14–15.9	0.0485	0	0.757	−0.2591 to 0.3560
≥16	0.1538	2	0.553	−0.3548 to 0.6623
Mean arterial pressure				
<80	1 (ref)	0	—	—
80–99	0.0395	0	0.880	−0.4736 to 0.5525
100–119	0.1324	1	0.606	−0.3710 to 0.6359
120–139	0.3417	3	0.225	−0.2099 to 0.8933
≥140	0.7527	8	0.027	0.0851 to 1.4203
No IVT	0.3844	4	0.002	0.1410 to 0.6279
Model 2 (early post-EVT): including model 1 + EVT-related variables				
EVT contrast volume				
0–100 mL	1 (ref)	0	—	—
100–299 mL	0.2624	3	0.074	−0.0260 to 0.5509
≥300 mL	0.5197	5	0.194	−0.2645 to 1.3039

Abbreviations: CA-AKI = contrast-associated acute kidney injury; EVT = endovascular thrombectomy; GFR = glomerular filtration rate; IVT = IV thrombolysis; NIHSS = NIH Stroke Scale.

Coefficients, integer scores, *p* values, and CIs for all pre-EVT variables are derived from model 1 (pre-EVT variables only). Estimates for EVT contrast volume are derived from the full model (model 2, including model 1 variables + EVT contrast volume). Results for pre-EVT variables in the full model were consistent with those in model 1, showing only minor variations and no changes in the assigned integer scores; therefore, they are not reported.

**Figure 3:**
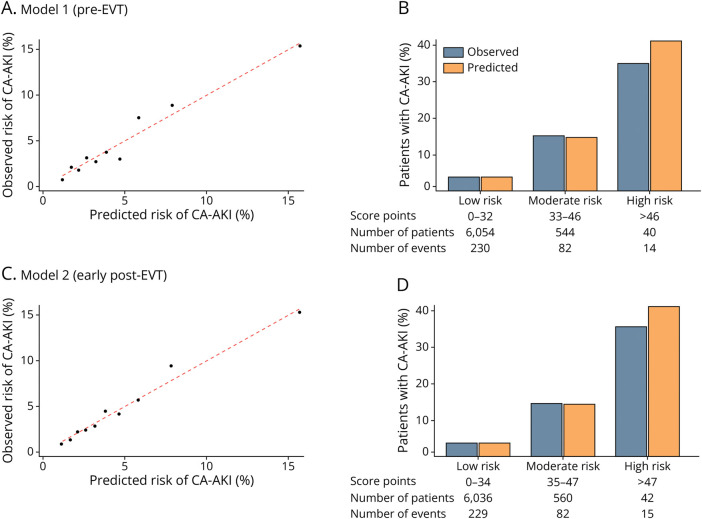
Calibration and Risk Stratification of CA-AKI Predictive Models Panels A and C: calibration plots showing observed vs predicted CA-AKI risk; the red dashed line indicates perfect calibration; model 1 (A) and model 2 (C). Panels B and D: observed vs predicted risk across predefined low-risk, moderate-risk, and high-risk strata; model 1 (B) and model 2 (D). CA-AKI = contrast-associated acute kidney injury; EVT = endovascular thrombectomy.

**Figure 4:**
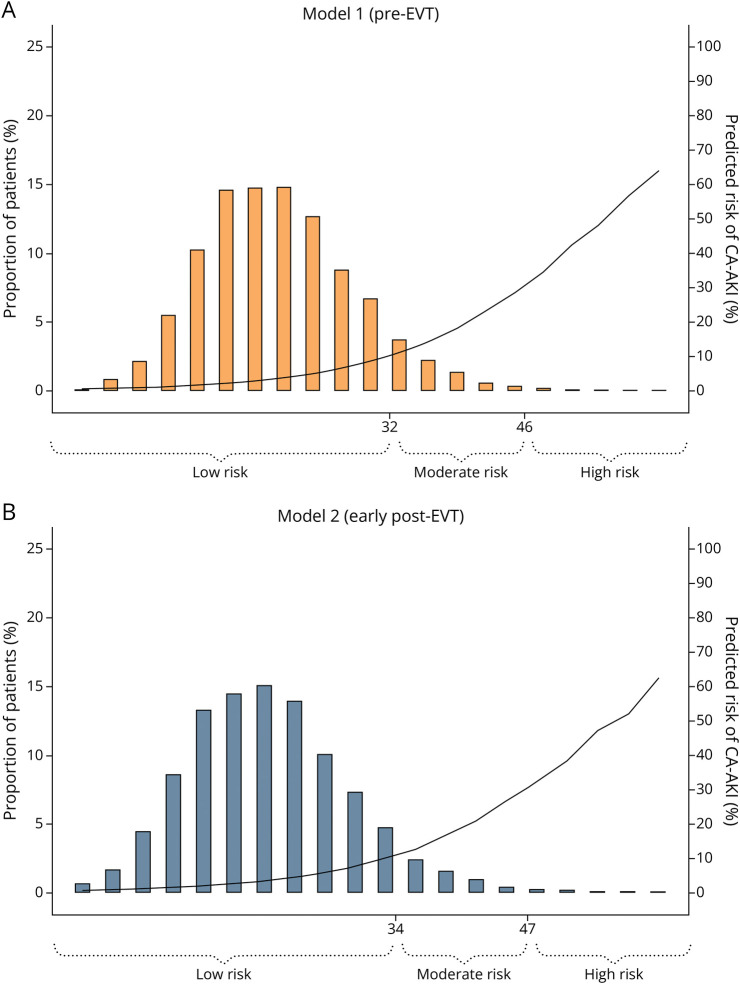
Risk Score Distribution and Model-Predicted CA-AKI Probability A (model 1, pre-EVT): x-axis, the risk score grouped into contiguous 3-point bins; left y-axis, the proportion of patients within each bin (bars); right y-axis, model-predicted CA-AKI probability (curve). Brackets indicate predefined low-risk, moderate-risk, and high-risk strata. B (model 2, early post-EVT): same layout as above; x-axis, the risk score in 3-point bins; left y-axis, the proportion of patients per bin; right y-axis, model-predicted CA-AKI probability (curve). Brackets indicate corresponding risk strata. CA-AKI = contrast-associated acute kidney injury; EVT = endovascular thrombectomy.

Figure 3 shows, in the full data set, the continuous calibration plots for both models, as well as the agreement between predicted and observed CA-AKI probabilities across the 3 risk categories (low, moderate, and high risk). Figure 4 further displays the distribution of risk scores and their corresponding model-predicted CA-AKI probabilities, highlighting the progressive increase in estimated risk across the predefined low-risk, moderate-risk, and high-risk strata.

## Discussion

In this large, multicenter cohort of patients undergoing EVT for AIS, we report 3 key findings. First, CA-AKI occurred in 4.9% of cases. Second, CA-AKI was independently associated with in-hospital mortality, as well as with worse functional outcome and increased mortality at 90 days. Third, we developed and internally validated 2 predictive models for CA-AKI, based on routinely available preprocedural and procedural variables, with acceptable discrimination and good calibration.

Although a direct causal role cannot be established, CA-AKI is a known complication of intravascular contrast use, with incidence rates ranging from 1% to 2% in low-risk settings to over 15% in high-risk populations, such as patients undergoing percutaneous coronary interventions.^9^ In the setting of AIS treated with EVT, the incidence of CA-AKI is variable, ranging from 2.5%^21^ to 12.6%,^22^ with a recent meta-analysis reporting a pooled incidence of approximately 7%.^13^ The heterogeneity in reported rates likely reflects the lack of a standardized diagnostic definition, with variability in both the magnitude of serum creatinine change (e.g., relative increase ≥25%, or absolute increase ≥0.3 or ≥0.5 mg/dL) and the time window for assessment (e.g., 48 hours vs 3, 5, or 7 days). We defined CA-AKI as an increase in serum creatinine ≥0.3 mg/dL, or ≥1.5 times baseline, within 48 hours after contrast administration, in line with KDIGO criteria.^15^ This definition aligns with recommendations for studies on contrast-associated renal impairment^23^ and helps minimize confounding from in-hospital events occurring beyond the acute phase (i.e., up to 7 days) that may independently affect renal function and influence outcomes.

Our findings confirm that preexisting renal dysfunction and lower baseline eGFR are the strongest predictors of CA-AKI, consistent with previous evidence showing increased vulnerability in patients with chronic kidney disease.^6,24,25^ In addition, acute-phase variables such as baseline stroke severity and admission hyperglycemia were independently associated with CA-AKI. While detailed mechanistic interpretation is beyond the scope of this study, a plausible hypothesis is that these factors may reflect the extent of systemic stress and metabolic dysregulation contributing to renal injury—mechanisms that have also been described in non-neurologic populations.^7^ Of interest, extreme values of baseline hemoglobin were also associated with CA-AKI risk. Although the underlying mechanisms remain unclear, higher levels may reflect dehydration and increased blood viscosity, whereas lower levels—also linked to CA-AKI in other scores,^8^ and often associated with advanced chronic kidney disease—could exacerbate renal hypoxic injury.

We found an association between contrast volume during EVT and the occurrence of CA-AKI. Although our study was not designed to establish causality—a topic of ongoing debate^4^—these results emphasize the need to consider contrast exposure as a relevant factor in stroke care, likely acting synergistically with other acute-phase factors. Our findings do not support withholding EVT or avoiding contrast during the procedure; rather, they warrant prudent use, close monitoring, and thorough documentation. Previous studies have shown that contrast exposure before EVT has minimal impact in patients with AIS.^26^ In line with these findings, we observed no association between pre-EVT contrast administration and CA-AKI. Targeted studies are needed to determine whether the discrepancy between pre-EVT and EVT-related exposure reflects cumulative dose effects and/or differences in route of administration, with intra-arterial delivery during EVT likely carrying greater nephrotoxic potential than IV injection.^12^

We also observed a disparity in CA-AKI susceptibility across ethnic groups. Although non-White patients comprised a small proportion of our cohort, most were Black, and this subgroup demonstrated a higher risk of CA-AKI. This finding is consistent with previous studies reporting increased susceptibility among Black patients undergoing intra-arterial contrast procedures.^27^ Such disparities, along with sex differences, warrant dedicated future studies to determine whether they reflect intrinsic biological or genetic susceptibility or, instead, residual and unmeasured confounding, including the potential role of structural and socioeconomic factors.

The inverse association we found between dyslipidemia and CA-AKI may reflect a protective effect of statins,^28^ which are commonly prescribed in this population. Statins have been proposed to mitigate CA-AKI risk through anti-inflammatory and antioxidant mechanisms.^7^ Indeed, in non-neurologic settings, preprocedural statin therapy has been shown to significantly reduce CA-AKI risk and improve clinical outcomes.^29,30^ Although statin use was not recorded in our data set, this association supports the need for targeted studies on statins for CA-AKI prevention in patients with AIS undergoing EVT.

The association between prestroke antiplatelet therapy and CA-AKI may reflect the presence of higher baseline vascular risk and greater clinical frailty in these patients. Moreover, the inverse association between IVT and CA-AKI observed in our cohort is consistent with previous reports^22^ and, although not conclusive, suggests a potential protective effect. While a direct renal mechanism cannot be excluded, it is plausible that IVT facilitates earlier recanalization, leading to shorter procedures and reduced contrast exposure, thereby indirectly mitigating the risk of kidney injury.

Finally, it is noteworthy that procedural hypotension, although significant in the univariate analysis and a key component of cardiology scores,^8^ was not retained in our model. This may reflect limited power due to the small number of events, differences in definition (our study defined hypotension as a blood pressure drop requiring inotropic support during EVT rather than a quantitative reduction in blood pressure), and potential confounding factors such as type of anesthesia during EVT, which was not available in the present data set. Nonetheless, the relevance of hemodynamic status in CA-AKI risk is supported by the independent association with baseline mean arterial pressure, which was included in the score.

Although CA-AKI occurred in a minority of patients, its clinical implications were substantial. Consistent with previous studies,^31^ affected patients experienced significantly worse functional outcomes and higher mortality, with a 90-day mortality rate approaching 50%. It is important to note that these associations remained significant even after adjusting for multiple variables known to be associated with worse outcomes (including, among the most relevant, stroke severity, comorbidities, occlusion site, onset-to-EVT time, EVT duration, number of passes, successful recanalization, and symptomatic hemorrhagic transformation), suggesting an independent effect. Multiple mechanisms may underlie this relationship. AKI can lead to systemic inflammation, fluid overload, and metabolic disturbances, all of which may impair brain recovery.^32^ Increasing interest has also been directed toward the kidney-brain axis, a bidirectional pathway in which renal dysfunction may contribute to cerebrovascular injury through inflammatory and metabolic mechanisms. This axis may be particularly relevant in the setting of CA-AKI, where acute renal injury could exacerbate systemic and neuroinflammatory responses, further affecting neurologic recovery.^33^ Moreover, AKI may lead to the interruption of essential poststroke therapies, prolong hospitalization with an increased risk of infectious complications, or delay rehabilitation due to medical instability, further compromising functional outcomes. Regardless of the exact mechanisms, CA-AKI clearly identifies a high-risk subgroup. These findings emphasize the need for early recognition and prevention.

In this context, we developed a simple, user-friendly risk score based on common clinical variables, allowing for bedside application and potential integration into electronic health records. The model using only pre-EVT variables (model 1) showed acceptable discrimination, and the inclusion of procedural data (model 2) did not significantly improve performance. This suggests that the score may be reliably applied even in the hyperacute phase, before procedural data are available, thereby optimizing the timing of potential preventive strategies. Despite its clinical relevance, CA-AKI remains underexplored in the setting of patients with AIS undergoing EVT. A recent meta-analysis including approximately 20 studies—albeit heterogeneous in design—consistently identified chronic kidney disease as the main risk factor and confirmed the association of CA-AKI with worse clinical outcomes.^13^ No score has been specifically developed or validated for patients with AIS undergoing EVT. Our study fills this gap by highlighting the prognostic relevance of CA-AKI in EVT-treated patients with AIS and providing a practical tool for early risk stratification. In the cardiology field, several predictive scores for CA-AKI have been developed in patients undergoing interventional procedures. However, these scores cannot be directly translated to the stroke population because they include variables that are specific to cardiology practice (e.g., left ventricular ejection fraction; clinical presentations such as stable angina, unstable angina, non–ST-segment elevation myocardial infarction, or ST-segment elevation myocardial infarction; periprocedural myocardial infarction; and the number and type of coronary lesions).^8^ Our stroke-specific risk models demonstrate acceptable discriminative ability, comparable to that of one of the most widely used CA-AKI scores in the cardiology field.^8^ The PR-AUC analysis was consistent with the receiver operating characteristic–based results, supporting the overall robustness of the model's discriminative performance despite the low event rate. While the presence of CA-AKI risk factors should not delay or preclude EVT in eligible patients, early identification of high-risk individuals may guide preventive strategies and optimize supportive care in this vulnerable population. In the near term, the CAN-REST score will be released as an online tool and externally validated in a temporally independent cohort, followed by clinical implementation and dissemination.

This study has several strengths. Based on a large-scale international multicenter cohort, it offers real-world evidence on a relatively uncommon but clinically relevant complication. A data set of this magnitude allows for the development of a reliable predictive score, supported by standardized data collection and rigorous methodology. Several limitations should nevertheless be acknowledged. First, the retrospective design may have introduced selection bias, despite efforts to minimize it through the inclusion of consecutive patients and standardized data collection procedures. A considerable proportion of cases had to be excluded because of missing follow-up creatinine measurements, which likely reflects real-world clinical practice, where early laboratory reassessment is often omitted in patients with either an uncomplicated clinical course or rapid deterioration. As a result, certain patient subgroups may be underrepresented in our analysis. While the study provides valuable insights grounded in routine stroke care, this limitation may affect the generalizability of the findings to the overall stroke population. Second, serum creatinine measurements were not collected according to a uniform protocol across centers, potentially leading to variability in the timing and sensitivity of CA-AKI detection. In addition, CA-AKI was defined according to KDIGO criteria, which, while ensuring consistency with international standards and clinical applicability, entails dichotomization of serum creatinine and a potential reduction in statistical power compared with modeling it as a continuous variable. Third, although we collected urine output at 24 hours after EVT, the KDIGO urine output criterion (<0.5 mL/kg/h for 6 hours) could not be applied because this definition requires continuous measurement over a fixed 6-hour window. A single cumulative 24-hour value, which was available retrospectively, does not allow reliable identification of transient or sustained oliguria and was, therefore, not used for AKI classification. Fourth, in 28.5% of patients, data on the amount of contrast media administered during EVT were missing and, therefore, imputed. This limitation likely reflects the limited emphasis currently placed on systematic contrast documentation—an issue that, in light of our findings, deserves greater attention in stroke care. While the type of contrast media (e.g., iso-osmolar vs low-osmolar) was available in most cases, information on iodine concentration (e.g., 300 vs 350 mg I/mL) was inconsistently reported. These physicochemical characteristics may influence contrast viscosity and nephrotoxic potential. Furthermore, individual patients may have received different contrast agents at multiple time points (e.g., during pre-EVT imaging and during the EVT procedure), limiting the ability to consistently classify overall contrast exposure. For these reasons, the impact of specific contrast media characteristics on CA-AKI risk was not explored and should be addressed in future studies. Similarly, the cumulative effect of contrast exposure (pre-EVT + EVT-related) was not assessed. This decision was based on the potential collinearity between the amounts of contrast administered before and during EVT and on the intention to evaluate preprocedural and intraprocedural exposure separately, given their potentially distinct clinical relevance. Fifth, we did not collect data on diuretic administration and on midterm to long-term renal function.

In this large, multicenter cohort of patients with AIS undergoing EVT, CA-AKI occurred in approximately 1 in 20 patients and was independently associated with worse functional outcomes and increased mortality. These findings underscore the prognostic relevance of this potentially preventable complication in the context of acute stroke care and highlight the importance of early identification. Although external validation is warranted, the CAN-REST predictive score developed and validated in this study provides a practical tool for identifying patients at risk of CA-AKI even before EVT and may support targeted prevention strategies in high-risk populations.

## Glossary

AIS: acute ischemic stroke
AKI: acute kidney injury
aOR: adjusted odds ratio
AUC: area under the receiver operating characteristic curve
CA-AKI: contrast-associated AKI
CAN-REST: Contrast-Associated Nephropathy Risk Evaluation in Acute Ischemic Stroke After Endovascular Thrombectomy
eGFR: estimated glomerular filtration rate
EVT: endovascular thrombectomy
IQR: interquartile range
IVT: IV thrombolysis
KDIGO: Kidney Disease: Improving Global Outcomes
mRS: modified Rankin Scale
PR-AUC: precision-recall area under the curve

## References

[1] Mettler FAJr, Mahesh M, Bhargavan-Chatfield M, et al. Patient exposure from radiologic and nuclear medicine procedures in the United States: procedure volume and effective dose for the period 2006-2016. Radiology. 2020;295(2):418-427. doi:10.1148/radiol.2020192256 32181730 PMC9754695

[2] Seeliger E, Sendeski M, Rihal CS, Persson PB. Contrast-induced kidney injury: mechanisms, risk factors, and prevention. Eur Heart J. 2012;33(16):2007-2015. doi:10.1093/eurheartj/ehr494 22267241

[3] Bartels ED, Brun GC, Gammeltoft A, GjØRup PA. Acute anuria following intravenous pyelography in a patient with myelomatosis. Acta Med Scand. 1954;150(4):297-302. doi:10.1111/j.0954-6820.1954.tb18632.x 13217726

[4] Vandenberghe W, Hoste E. Contrast-associated acute kidney injury: does it really exist, and if so, what to do about it? F1000Res. 2019;8:F1000 Faculty Rev-753. doi:10.12688/f1000research.16347.1 PMC6544074 31275558

[5] Solomon R Contrast-induced acute kidney injury (CIAKI). Radiol Clin North Am. 2009;47(5):783-788, v. doi:10.1016/j.rcl.2009.06.001 19744593

[6] Davenport MS, Perazella MA, Yee J, et al. Use of intravenous iodinated contrast media in patients with kidney disease: consensus statements from the American College of Radiology and the National Kidney Foundation. Radiology. 2020;294(3):660-668. doi:10.1148/radiol.2019192094 31961246

[7] Mehran R, Dangas GD, Weisbord SD. Contrast-associated acute kidney injury. N Engl J Med. 2019;380(22):2146-2155. doi:10.1056/NEJMra1805256 31141635

[8] Mehran R, Owen R, Chiarito M, et al. A contemporary simple risk score for prediction of contrast-associated acute kidney injury after percutaneous coronary intervention: derivation and validation from an observational registry. Lancet. 2021;398(10315):1974-1983. doi:10.1016/S0140-6736(21)02326-6 34793743

[9] Faggioni M, Mehran R. Preventing contrast-induced renal failure: a guide. Interv Cardiol Rev. 2016;11(2):98-104. doi:10.15420/icr.2016:10:2 PMC5808627 29588714

[10] GBD 2021 Stroke Risk Factor Collaborators. Global, regional, and national burden of stroke and its risk factors, 1990-2021: a systematic analysis for the Global Burden of Disease Study 2021. Lancet Neurol. 2024;23(10):973-1003. doi:10.1016/S1474-4422(24)00369-7 39304265 PMC12254192

[11] Goyal M, Menon BK, van Zwam WH, et al. Endovascular thrombectomy after large-vessel ischaemic stroke: a meta-analysis of individual patient data from five randomised trials. Lancet. 2016;387(10029):1723-1731. doi:10.1016/S0140-6736(16)00163-X 26898852

[12] Schönenberger E, Martus P, Bosserdt M, et al. Kidney injury after intravenous versus intra-arterial contrast agent in patients suspected of having coronary artery disease: a randomized trial. Radiology. 2019;292(3):664-672. doi:10.1148/radiol.2019182220 31264950

[13] Oliveira M, Rocha A, Barbosa F, et al. Acute kidney injury after endovascular therapy in acute stroke patients: systematic review with meta-analysis. J Neurointerv Surg. 2023;15(e3):e468-e474. doi:10.1136/jnis-2022-019955 36797049

[14] Collins GS, Moons KGM, Dhiman P, et al. TRIPOD+AI statement: updated guidance for reporting clinical prediction models that use regression or machine learning methods. BMJ. 2024;385:e078378. doi:10.1136/bmj-2023-078378 38626948 PMC11019967

[15] Khwaja A. KDIGO clinical practice guidelines for acute kidney injury. Nephron Clin Pract. 2012;120(4):c179-c184. doi:10.1159/000339789 22890468

[16] Colin Cameron A, Miller DL. A practitioner's guide to cluster-robust inference. J Hum Resour. 2015;50(2):317-372. doi:10.3368/jhr.50.2.317

[17] Riley RD, Snell KI, Ensor J, et al. Minimum sample size for developing a multivariable prediction model: part II: binary and time-to-event outcomes. Stat Med. 2019;38(7):1276-1296. doi:10.1002/sim.7992 30357870 PMC6519266

[18] Pavlou M, Ambler G, Qu C, Seaman SR, White IR, Omar RZ. An evaluation of sample size requirements for developing risk prediction models with binary outcomes. BMC Med Res Methodol. 2024;24(1):146. doi:10.1186/s12874-024-02268-5 38987715 PMC11234534

[19] Saito T, Rehmsmeier M. The precision-recall plot is more informative than the ROC plot when evaluating binary classifiers on imbalanced datasets. PLoS One. 2015;10(3):e0118432. doi:10.1371/journal.pone.0118432 25738806 PMC4349800

[20] Steyerberg EW, Vickers AJ, Cook NR, et al. Assessing the performance of prediction models: a framework for traditional and novel measures. Epidemiology. 2010;21(1):128-138. doi:10.1097/EDE.0b013e3181c30fb2 20010215 PMC3575184

[21] Laible M, Jenetzky E, Möhlenbruch MA, Bendszus M, Ringleb PA, Rizos T. The impact of post-contrast acute kidney injury on in-hospital mortality after endovascular thrombectomy in patients with acute ischemic stroke. Front Neurol. 2021;12:665614. doi:10.3389/fneur.2021.665614 34163423 PMC8215575

[22] Wrona P, Sawczyńska K, Wróbel D, et al. Risk factors of acute kidney injury during hospitalization in acute ischaemic stroke patients undergoing mechanical thrombectomy. Postepy Kardiol Interwencyjnej. 2024;20(1):89-94. doi:10.5114/aic.2024.136374 38616933 PMC11008514

[23] van der Molen AJ, Reimer P, Dekkers IA, et al. Post-contrast acute kidney injury: part 1: definition, clinical features, incidence, role of contrast medium and risk factors: recommendations for updated ESUR Contrast Medium Safety Committee guidelines. Eur Radiol. 2018;28(7):2845-2855. doi:10.1007/s00330-017-5246-5 29426991 PMC5986826

[24] Weber R, van Hal R, Stracke P, Hadisurya J, Nordmeyer H, Chapot R. Incidence of acute kidney injury after computed tomography angiography ± computed tomography perfusion followed by thrombectomy in patients with stroke using a postprocedural hydration protocol. J Am Heart Assoc. 2020;9(4):e014418. doi:10.1161/JAHA.119.014418 32067579 PMC7070223

[25] Wang R, Xie Z, Li B, Zhang P. Renal impairment and the prognosis of endovascular thrombectomy: a meta-analysis and systematic review. Ther Adv Neurol Disord. 2022;15:17562864221083620. doi:10.1177/17562864221083620 35646161 PMC9133867

[26] Brinjikji W, Demchuk AM, Murad MH, et al. Neurons over nephrons: systematic review and meta-analysis of contrast-induced nephropathy in patients with acute stroke. Stroke. 2017;48(7):1862-1868. doi:10.1161/strokeaha.117.016771 28583996

[27] Lunyera J, Clare RM, Chiswell K, et al. Racial differences in AKI incidence following percutaneous coronary intervention. J Am Soc Nephrol. 2021;32(3):654-662. doi:10.1681/asn.2020040502 33443096 PMC7920184

[28] Marenzi G, Cosentino N, Werba JP, Tedesco CC, Veglia F, Bartorelli AL. A meta-analysis of randomized controlled trials on statins for the prevention of contrast-induced acute kidney injury in patients with and without acute coronary syndromes. Int J Cardiol. 2015;183:47-53. doi:10.1016/j.ijcard.2015.01.046 25662053

[29] Quintavalle C, Fiore D, De Micco F, et al. Impact of a high loading dose of atorvastatin on contrast-induced acute kidney injury. Circulation. 2012;126(25):3008-3016. doi:10.1161/circulationaha.112.103317 23147173

[30] Leoncini M, Toso A, Maioli M, et al. Early high-dose rosuvastatin and cardioprotection in the protective effect of rosuvastatin and antiplatelet therapy on contrast-induced acute kidney injury and myocardial damage in patients with acute coronary syndrome (PRATO-ACS) study. Am Heart J. 2014;168(5):792-797. doi:10.1016/j.ahj.2014.08.005 25440809

[31] Oliveira M, Sousa M, Antunes R, et al. Early acute kidney injury in stroke patients submitted to endovascular treatment: a cohort study. J Clin Med. 2024;13(22):6726. doi:10.3390/jcm13226726 39597868 PMC11594989

[32] Zhao Q, Yan T, Chopp M, Venkat P, Chen J. Brain–kidney interaction: renal dysfunction following ischemic stroke. J Cereb Blood Flow Metab. 2020;40(2):246-262. doi:10.1177/0271678x19890931 31766979 PMC7370616

[33] Kelly DM, Kelleher EM, Rothwell PM. The kidney-immune-brain axis: the role of inflammation in the pathogenesis and treatment of stroke in chronic kidney disease. Stroke. 2025;56(4):1069-1081. doi:10.1161/STROKEAHA.124.047070 39851054 PMC11932449

